# Medical Device Sterilization and Reprocessing in the Era of Multidrug-Resistant (MDR) Bacteria: Issues and Regulatory Concepts

**DOI:** 10.3389/fmedt.2020.587352

**Published:** 2021-02-10

**Authors:** Jonathan Josephs-Spaulding, Om V. Singh

**Affiliations:** ^1^Department of Environmental Health and Engineering, Johns Hopkins Bloomberg School of Public Health, Baltimore, MD, United States; ^2^Advance Academic Program, The Johns Hopkins University, Washington, DC, United States; ^3^Technology Science Group (TSG) Consulting Inc., A Science Group Company, Washington, DC, United States

**Keywords:** multidrug-resistant (MDR) bacteria, medical devices, sterilization, reprocessing, regulation, US FDA, biofilms

## Abstract

The emergence of multidrug-resistant (MDR) bacteria threatens humans in various health sectors, including medical devices. Since formal classifications for medical device sterilization and disinfection were established in the 1970's, microbial adaptation under adverse environmental conditions has evolved rapidly. MDR microbial biofilms that adhere to medical devices and recurrently infect patients pose a significant threat in hospitals. Therefore, it is essential to mitigate the risk associated with MDR outbreaks by establishing novel recommendations for medical device sterilization, in a world of MDR. MDR pathogens typically thrive on devices with flexible accessories, which are easily contaminated with biofilms due to previous patient use and faulty sterilization or reprocessing procedures. To prevent danger to immunocompromised individuals, there is a need to regulate the classification of reprocessed medical device sterilization. This article aims to assess the risks of improper sterilization of medical devices in the era of MDR when sterilization procedures for critical medical devices are not followed to standard. Further, we discuss key regulatory recommendations for consistent sterilization of critical medical devices in contrast to the risks of disinfection reusable medical devices.

## Introduction

**Figure 1:**
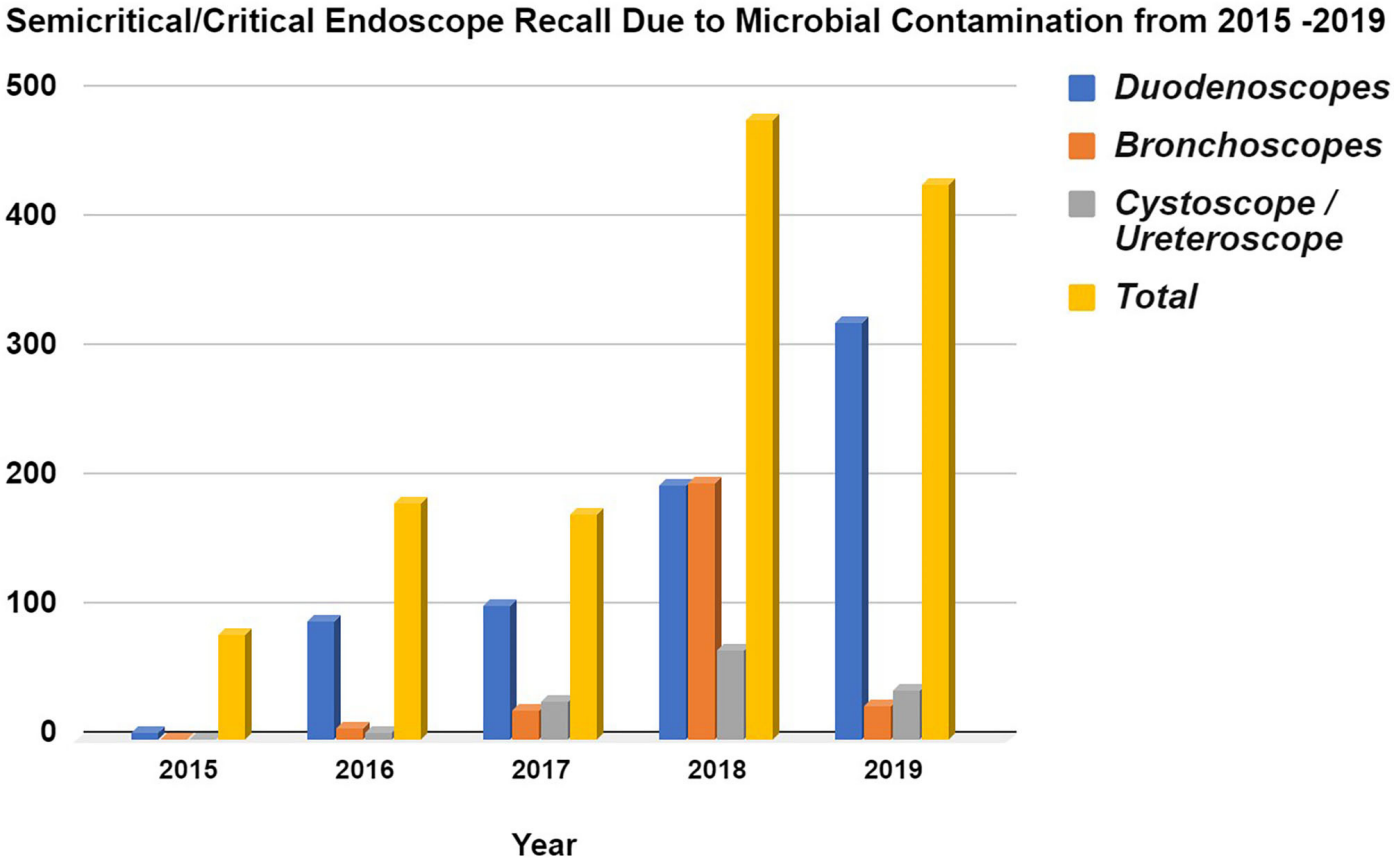
Semi-critical/Critical Endoscope Voluntary Recall due to Microbial Contamination from 2015 to 2019 based from the FDA's MAUDE database. Data acquired from Manufacturer and User Facility Device Experience using product problem “Microbial contamination of devices” under product classes, (i). Duodenoscope and Accessories, Flexible/Rigid, (ii). Bronchoscope & Accessories, Flexible/Rigid, (iii). Cystoscope & Accessories, Flexible/Rigid. The data was collected for every year since 2015 onwards (https://www.accessdata.fda.gov/scripts/cdrh/cfdocs/cfMAUDE/Search.cfm?smc=1).

Within the framework of the United States Food Drug & Cosmetics Act (FD&C Act), a medical device is defined as “An instrument apparatus, implement, machine, contrivance, implant, *in vitro* reagent, or other similar or related article, including a component part or accessary which is intended for use in the diagnosis of disease or other conditions, or in the cure, mitigation, treatment or prevention of disease in man or other animals, or intended to affect the structure or any function or other animals.” The sterilization of a medical device is the chemical or physical process of eliminating all forms of microbial life and associated spores through a variety of methods, such as autoclaving with heat/pressure, hydrogen peroxide vapor, radiation, ethylene oxide (EtO) gas, and other processes. It is essential to control the risk of any procedure involving semi-critical or critical medical devices that may be deleterious to patient health (1, 2). Figure 1 summarizes the FDA's Manufacturer and User Facility Device Experience (MAUDE), illustrating the increased recall of both semi-critical and critical medical devices (duodenoscope, bronchoscope, cystoscope/ureteroscope) due to microbial contamination between the years 2015–2019.

Infection in patients groups at low risk of contracting infectious diseases from medical devices are rapidly increasing. This is due to the increased prevalence of multidrug-resistant (MDR) pathogens with biofilm-forming potential. These contagious agents often harbor genomic resistance to a wide-spectrum antibiotics, in addition to last-resort antibiotics such as carbapenems. Although, the true incidence of health care-associated burden of infections from medical devices is likely under-reported due to lack of surveillance or the absence of clinical manifestation (3–7). Therefore, sterilization of medical devices is essential to prevent MDR outbreaks and control hospital-acquired nosocomial infections.

**Figure 2:**
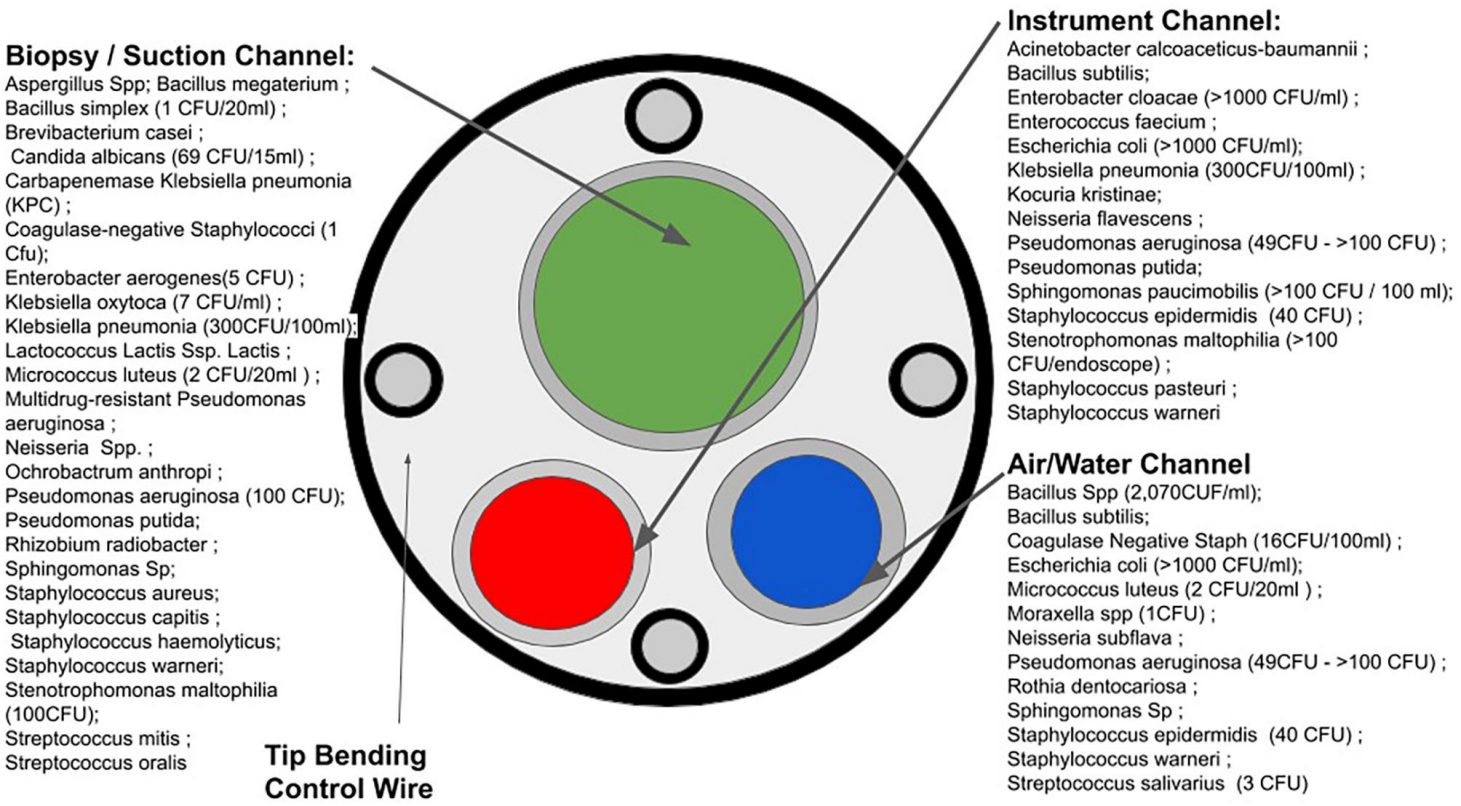
Microbial susceptible sites of endoscopes and microbial contamination. (Based on the data acquired in Figure 1 from the FDA's MAUDE database).

Due to high financial costs associated with single-use critical medical devices, it is tempting to repurpose single-use products as single-patient (8): rather than using a device only once, practitioners may use the device multiple times over hours or days on the same patient. This practice also bypasses some time-consuming standards for high-level disinfection (9). In one reported case, potentially contaminated medical devices were used on various patients to maintain the status quo of duodenoscopy procedures in a hospital (10). Failure to properly sterilize critical medical devices for various endoscopy procedures such as bronchoscopy, cystoscopy, and duodenoscopy has led to numerous outbreaks of MDR pathogens with serious consequences to patients (11–13). Figure 2 illustrates a variety of sites on endoscope devices that are susceptible to microbial contamination, and the diversity of microbes which occupy this space.

Due to the rapid spread and development of MDR bacteria in hospital environments, there is a need to update regulatory guidelines to reflect the modern evolution of microbiota. However, these shifts require additional efforts to explore the effective means of sterilization for a wider range of semi-critical and critical medical devices. The presented article aims to discuss the risks of improper sterilization of medical devices, given the facts of rapid microbial evolution and development of biofilms to resist disinfection in hospital settings (14). Furthermore, the key regulatory recommendations in this article would ensure the benefits of consistent sterilization of critical medical devices, in contrast to the risks of reprocessing or reusing devices without high sterilization protocols.

## Medical Devices and Sterilization

Procedures that breach sterile tissue or areas or mucous membranes in patient, involves different levels of invasive procedures. The level of invasion potential defines the risk of infection caused by the device and the appropriate microbicidal processes to use (15, 16). Based on the degree of invasion, medical devices are classified as critical or semi-critical. Because the Spaulding classification does not address all clinical devices and their intended uses, the US Food and Drug Administration (FDA) has modified the categories to critical, semi-critical, and non-critical depending on their intended use (17).

Critical devices are those introduced directly into the bloodstream or in contact with body spaces, including sterile tissues. These include most surgical instruments; irrigation systems for sterile instruments in sterile tissues; endoscopes used in sterile body cavities such as laparoscopes, arthroscopes, and intravascular endoscopes; and all endoscope biopsy accessories (17).

Devices that come into contact with intact mucous membranes or non-intact skin of the patient are classified as semi-critical devices. These devices include duodenoscopes, endotracheal tubes, bronchoscopes, laryngoscope blades and other respiratory equipment, esophageal manometry probes, diaphragm fitting rings, and gastrointestinal endoscopes (17).

Any device that contacts only intact skin and does not penetrate into the skin is classified under non-critical devices. These devices include blood pressure cuffs, stethoscopes, skin electrodes, etc. However, some of these devices, such as infusion pumps and ventilators, may still become contaminated during patient care despite their classification as non-critical (17).

Depending on the indicative use leading to the classification of a medical device, cleaning and sterilization strategies may vary. Sterilization procedures of medical devices require elimination of viable microbiota in a timely and efficient fashion. The original hierarchy developed by Spaulding was based on microbial resistance to various disinfection processes; microbes have drastically evolved to resist human approaches to their elimination. Therefore, the practice and regulations surrounding critical medical devices per the FDA's guidance must incorporate changes in the classification of medical devices for sterilization, that mirror evolutionary changes in MDR bacteria. One significant concern is that FDA guidance does not cover the occurrence of MDR. Rather, the FDA directs medical institutes to follow guidance from the Centers for Disease Control (18). There is a dire need to harmonize strategies to mitigate MDR biofilms on medically significant devices across regulatory and professional bodies (19, 20).

## Terminal Sterilization and Looming Threats of MDR

MDR is a looming global threat to both patient safety and public health. MDR occurs through the evolution of microbiota exposed to sub-lethal levels of antibiotics without eliminating all populations of pathogens. Surviving strains regrow and pass on genes that enable microbiota to survive drugs such as β-lactam antibiotics (through the expression of metallo-β-lactamase enzymes) or carbapenem (a last-resort antibiotic) (21–23). While device-related occurrences of MDR are infrequent, devices with flexible mechanics that interact with the microbially abundant GI system, and the bronchioles or urogenital system which are less microbially diverse, carry a high risk of infecting patients when regulations for disinfection or sterilization are not followed (24). For example, flexible GI endoscopes or duodenoscopes include a complex design of interconnecting channels and elevator shaft mechanisms within the channels. These intricate parts are necessary for controlling fine movements that may be different for each specific procedure (25). This characteristic of flexible duodenoscopes is a double-edged sword that provides a challenge for device sterilization. The internal areas of duodenoscopes may promote the growth of MDR pathogens and biofilms that resist both drugs and typical sterilization processes (26, 27).

A major issue overshadowing the need for terminally sterilized medical devices is the lack of global compliance with procedures for the cleaning and sterilization of semi-critical or critical medical devices. Generally, traditional practices for manual cleaning and disinfection alone are suboptimal to control hospital-associated infections. Only 40–50% of the surfaces that ought to be cleaned are actually cleaned by housekeepers to maintain a hygienic environment (28). This may be due to the hospitals' inability to enforce stringent recommendations set by regulatory agencies or the activity of MDR microbiota that resist such stringent protocols for cleaning and sterilization.

A double-blind study of 14 different hospitals identified that ~57% of sterilization processing department workers are not fully qualified to undertake critical sterilization of medical devices (29). In the same study, ~56% of 23 surveyed surgical devices were found to be contaminated following reprocessing (29). Additionally, a survey of 372 medical practitioners (90% practice nurses, 10% general practitioners) found that 14% of the surveyed group did not employ an autoclave between patients and only 55% of respondents had received detailed training in the prevention of cross-infections (30). Another study identified that during the reprocessing of orthopedic screws and plates, there was an increased presence of both carbohydrate residues and endotoxins on the medical devices (31). Therefore, in recent years, the safety and applicability of standard repurposing for reusable medical devices such as duodenoscopes has been heavily questioned. This is especially true when MDR outbreaks are associated with flexible duodenoscopes (32, 33).

These observations support the fact that medical staff lack proper training to handle semi-critical and critical medical devices. Due to technological advancements, the complexity of medical devices has increased in recent years; devices have become more intricate and many include small crevices for the addition of various accessories that require experts to reprocess devices prior to use. Numerous manufacturers are now moving toward providing critical or complicated medical devices as terminally sterile. This approach will strengthen patient safety and reduce hospital burden; manufacturers will benefit from terminal sterilization while maximizing the efficacy of medical devices. Terminal sterilization is a sound solution given the facts of: modern surgical suites, sudden or unplanned patient arrival in emergency rooms, poor logistics across medical care facilities, and the high volume of work required to maintain sterilization facilities. Table 1 provides a comprehensive overview of microbiota associated recalls of medical devices between the years 2015 and 2019, as described by the FDA's MAUDE database.

**Table 1:** Known bacteria of microbial contamination of semi-critical and critical medical devices which caused a recall during 2015–2019^*^.

**Cystoscope/ ureteroscope**	*Actinomyces Oris* (10 CFU/100 M*)* *Bacillus spp. mesophiles* (25 CFU/endoscope) *Comamonas testosteroni* (3 CFU/endoscope) *Corynebacterium glaucum* *Cryptococcus uniguttulatus* *Enterobacter cloacae* (3 CFU/100 ml) *Escherichia coli* *Haemophilus sputorum* *Klebsiella* (multidrug resistance) *Kocuria rhizophila* *Micrococcus luteus* (2 CFU); *M. lylae* *Moraxella osloensis* (1 CFU/endoscope) *Neisseria sp*. *Ochrobactrum anthropi* *Paenibacillus pabuli* *Pseudomonas aeruginosa; P. fluorescens* (>150 CFU/100 ml); *P. putida* (5 CFU/ 100 mL) *Staphylococcus aureus* (1 CFU/ 100 ml); *S. capitis*; *Staphylococcus* Coagulase Negative (1 CFU/Endoscope); *S. caprae* (1 CFU/100 mL); *S. epidermidis* (30 CFU); *S. haemolyticus*; *S. hominis* (1 CFU); *S. Lugdunensis* (3 CFU/150 mL); *S. pasteuri* (1 CFU/Channel); *S. saprophyticus; S. sciuri; S. warneri* (3 CFU/mL); *Stenotrophomonas maltophilia* (29 CFU/100 mL) *Streptococcus* spp.
**Duodenoscope**	*Acinetobacter baumannii-calcoaceticus; A. denitrificans* (40 CFU/100 ml); *A. johnsonii; A. kocuria; A. pittii*; *A. radioresistens; A. variabilis; A. caviae; A. hydrophila* *Aerococcus viridans* (7 CFU) *Bacillus altitudinis*; *B. aerophilus*; *B. aryabhattai;B. cereus; B. circulans; B. halodurans okuhiden; B. idriensis*; *B. oleovorans; B. megaterium/aryabhattai;* *B. simplex* (1 CFU/ 20 mL); *B. subterraneus; B. subtilis; B. thermoamylovorans; B. thermoamylovorans; B. thuringiensis;* *Brevibacterium casei* *Brevundimonas diminuta* (76 CFU/endoscope); *B. intermedia; B. nasdae*; *B. vesicularis* *Burkholderia cepacia* *Candida albicans* (69 CFU/15 mL); *C. glabrata; C. guilliermondii*; *C. parapsilosis; C. tropicalis* *Carbapenemase Klebsiella Pneumonia* (KPC) *Carbapenem-resistant Enterobacteriaceae* (CRE) *Cellulomonas pakistanensis* *Chryseobacterium indologenes; C. haifense* *Citrobacter freundii* (125 CFU) *Clostridium perfringens* Coagulase negative *Staph* (16 CFU/100 mL) *Corynebacterium aurimucosum/minutissimum*; *C. jeikeium*; *C. massiliensis*; *C. singular; C. striatum; C. tuberculostearicum* *Cupriavidus metallidurans* *Curtobacterium flaccumfaciens* *Deinococcus wulumuqiensis* *Delftia acidovorans* *Dermabacter hominis; D. vaginalis* *Dermacoccus nishinomiyaensis* *Dietzia papillomatosis* *Enterobacter aerogenes* (5 CFU); *E. cloacae* (>1,000 CFU/mL); *E. gergoviae; E. mori* *Enterococcus casseliflavus; E. durans*; *E. faecium; E. casseliflavus* (1 CFU); *E. faecalis; E. gallinarum* *Erwinia billingiae* *ESBL E. coli; E. coli* (>1,000 CFU/mL); *E. fergusonii; E. hermannii*; *E. dermatitidis* *Gemella Haemolysans* *Hamigera avellaneda* *Janibacter indicus* *Klebsiella oxytoca* (7 CFU/ mL); *K. pneumonia* (300 CFU/100 mL) *Kocuria koreensis; K. rhizophila* (1 CFU) *Kytococcus Schroeteri* *Leclercia Adecarboxylata* *Lysinibacillus* spp. *Massilia* sp. *MDR Pseudomonas aeruginasa* *Microbacterium lacus; M. paraoxydans* *Micrococcus luteus* (2 CFU/ 20 mL) *Moraxella* spp. (1 CFU) *Neisseria flavescens; N. subflava* *Neosartorya fischeri* *Ochrobactrum anthropi* *Paenibacillus lautus* *Paenibacillus provencensis* *Pantoea agglomerans* *Paracoccus yeei* *Pluralibacter Pyrinusafter* *Proteus mirabilis* *Pseudomonas aeruginosa* (49 CFU - >100 CFU); *P. Luteola* (>100 CFU/endoscope) *Pseudoxanthomonas mexicana* *Pseudomonas moorei; P. oryzihabitans; P. putida* (>100 CFU); *P. stutzeri* *Ralstonia* spp. *Raoultella ornithinolytica* *Rhizobium radiobacter* *Roseomonas mucosa* *Rothia dentocariosa; R. mucilaginosa* *Serratia Ureilytica* *Shigella dysenteriae* *Skermanella areolata* *Sphingomonas mucosissima; S. paucimobilis* (>100 CFU/100 mL) *Staphylococcus* sp.; *S. aureus*; *S. capitis; S. coagulase;* *S. cohnii* Subsp. *Urealyticum*; *S. epidermidis* (40 CFU); *S. haemolyticus; S. hominis* (2 CFU/channel); *S. lugdunensis; S. pasteuri* (4 CFU/endoscope); *S. petrasii; S. pettenkoferi; S. pseudolugdunensis; S. saprophyticus; S. warneri; S. agalactiae; S. dysgalactiae; S. mitis; S. oralis; S. parasanguinis; S. salivarius* (3 CFU); *S. sanguinis; S. viridans* *Stenotrophomonas maltophilia* (>100 CFU/endoscope) *Vancomycin Resistant (VRE); Enterococcus faecium* *Viridians streptococcus* *Xanthomonas maltophilia*
**Bronchoscope**	*Acinetobacter baumannii* *Actinomyces* spp. *Aspergillus s*pp. *Bacillus licheniformis; B. megaterium; B. niabensis* *Bacillus subtilis* *Candida glabrata* (3 CFU/plate); *C. gravulata; C. parapsilosis* (1 CFU) *Carbapenemase Klebsiella pneumonia* *Cellulosimicrobium cellulans* *Achromobacter xylosoxidans* (>100 CFU) *Chrseobacter* spp. *Chryseobacterium indologenes* Coagulase-negative *Staphylococci* (1 CFU) *Comamonas testosteroni* *Enterobacter aerogenes* (>100 CFU/ Endoscope); *E. cloacae* *Enterococcus casseliflavus* *Escherichia coli* *Klebsiella pneumoniae* (>10 CFU/mL) *Kocuria kristinae* *Lactococcus lactis* Sub-sp. *lactis* *Legionella* spp. *Geotrichum candidum* *Methylobacterium mesophilicum* *Microbacterium aurum* *Micrococcus luteus* (1 CFU/mL) *Multidrug-resistant Pseudomonas aeruginosa (MDRP)* *Mycobacterium kansasii; M. lentiflavum; M. Peregrinum; M. tuberculosis* *Neisseria mucosa* (2 CFU); *N subflava* *Paracoccus yeei* *Paracoccus warneri* *Pseudomonas aeruginosa; P. alcaligenes; P. putida* *Serratia marcescens* (red wild) *Shigella* Spp.
	*Staphylococcus aureus; S. capitis; S. epidermidis* (40 CFU); *S. haemolyticus; S. pasteuri; S. warneri; S. warneri*; *S. mitis; S. salivarius; S. viridans* *Stenotrophomonas maltophilia*
**Species in common**	*Escherichia coli*; *Pseudomonas aeruginosa*; *Staphylococcus aureus*; *S. epidermidis; S. warneri; Stenotrophomonas maltophilia*

* *Based on the data acquired in Figure 1 from the FDA's MAUDE database*.

## Regulatory Concepts for Medical Device Sterilization

Maintenance of sterile techniques is a key requirement for any surgical procedure. Common means of sterilization include dry heat, EtO, formaldehyde, gas plasma, peracetic acid, electron beams, and gamma rays. In the US, the FDA notes that the sterilization methods used in device manufacturing settings are subject to Quality System (QS) regulations per 21 CFR Part 820. To maintain the QS, the FDA considers two major categories of sterilization methods for medical device sterilization in manufacturing settings: established methods and novel methods.

Established sterilization methods are further divided into two categories: A and B. The Category A methods have a long history of safe and effective use for medical device sterilization using dry heat, EtO, steam, and radiation (e.g., gamma, electron beam). There are voluntary consensus standards for development, validation, and routine control that are recognized by the FDA (https://www.accessdata.fda.gov/scripts/cdrh/cfdocs/cfStandards/search.cfm).

The Category B methods lack FDA-recognized consensus standards, but published information on development, validation, and routine controls are recognized by the FDA. Based on the FDA's evaluation of validated data from the sterilizers, Category B methods include sterilization using hydrogen peroxide (H_2_O_2_), ozone (O_3_), and flexible bag systems (e.g., EtO in a flexible bag system, diffusion method, injection method).

Any sterilization method that has not been reviewed and determined to effectively sterilize devices for their intended use is classified as a novel method by the FDA. For example, any combination of chemicals that has not been reviewed, cleared, or approved by the FDA as a sterilant is considered a novel ingredient for the sterilization process. Other examples of novel methods include vaporized peracetic acid, high-intensity light or pulse light, microwave radiation, sound waves, and ultraviolet-light-mediated methods.

Per FDA guidance, a medical device labeled as sterile is required to meet with set standards to ensure that the device is safe and effective for its intended use in the US (34). However, there are no indications regarding extremophiles or MDR in the guidance document.

## Contamination and Persistence of Biofilms on Critical Medical Devices

MDR biofilms have the potential to contaminate and maintain colonies on a variety of critical-level medical devices, which foreshadow a grave future. The formation of biofilms and their high degree of tolerance to common antibiotics and sterilization methods is of the utmost public health significance. Understanding the dynamic interactions between complex microbial biofilm communities and their associations with both the human biotic and medical device abiotic environments is necessary to prevent MDR biofilms from developing on critical medical devices (14). Table 2 describes the diversity of microbiota that have been known to produce biofilms on medical devices.

**Table 2:** Diversity of biofilm producing bacteria on medical devices.

**Pathogen**	**Medical device**	**Source**
*Candida albicans, E. faecalis*	Flexible endoscope	(35)
*Candida parapsilosis*	Intravascular & prosthetic devices	(36)
*Carbapenem-resistant P. aeruginosa*	Endoscope	(4)
*Propionibacterium acnes*	Heart valve prosthesis	(37)
*P. aeruginosa*	Ureteroscope	(38)
*P. putida, Stenotrophomonas maltophilia*	Bronchoscope	(39)
*S. aureus, S. epidermidis*	Central venous catheters	(40)

Growth of biofilms on medical devices can occur through environmental contamination in a hospital setting. For example, the use of contaminated water supplies for routine cleaning of medical devices can lead to colonization and microbial growth on critical medical devices that were previously sterilized (41). Pathogens that form biofilms may also be transmitted from the skin of patients or healthcare workers and contaminate sterile objects in a healthcare facility. This form of transmission has been identified in the case of the human-associated *Staphylococcus epidermidis* and *Staphylococcus aureus* (42). Both are major pathogens that are able to form biofilms on medical devices. Some hypothesize that *S. epidermidis* may account for ~80% of bacteria involved in medical device infections (43, 44). In addition, both pathogens have caused recalls of critical medical devices (duodenoscopes, bronchoscopes, and cystoscopes) as described in Table 3.

**Table 3:** Reprocessing failure of MDR pathogens contaminating semi-critical or critical medical devices.

**Location or Institute**	**Year**	**Reason**	**Bacteria**	**Instrument involved**	**# of persons exposed**	**Clinical outcome**	**Source**
France	2008–2009	Contaminated endoscope channels, insufficient drying	ESBL-producing *K. pneumoniae*	Endoscope	16	Bacteremia/sepsis, cholangitis	(45)
France	2009	Contaminated endoscope channels, insufficient drying	KPC-producing *K. pneumoniae*	N/A	7	Bacteremia/sepsis, cholangitis	(46)
Netherlands	2008	Biofilm in endoscope channel	Carbapenem Resistant *Pseudomonas aeruginosa*	Endoscope	3	NA	(4)
Puerto Rico	2008	Improper cleaning of elevator shaft	*K. pneumoniae* (Carbapenem-resistant)	Endoscope	26	NA	(47)
Tampa, Florida	2008–2009	Bio-debris under elevator shaft	Carbapenemase-producing *Klebsiella pneumoniae*	Endoscope	7	1 death, 6 infections	(48)
Taiwan	2010	Contamination of video camera head	NDM-1 *K. pneumoniae*	Urological instrument	15	Fever and flank pain	(49)
Reimes, France	2011	Breaches in manual cleaning|wear of adhesive at the gastroscope's distal sheath	ESBL-producing *P. aeruginosa*	Gastroscope	4	NA	(50)
Germany	2012–2013	Imperfect disinfection	*K. pneumoniae* CP (OXA-48)	Duodenoscope	12	Bacteraemia/sepsis, pulmonary infections, surgical infection	(51)
Washington, USA	2013	Critical defects requiring repair, leak test failure	AmpC *Escherichia coli*	Duodenoscope	32	19 deaths	(33)
Oslo, Norway	2013	Heat-resistant pathogens	clpK *K. pneumoniae*	Endoscope and Bronchoscope	5	Pneumonia, sepsis, multi-organ failure	(52)
Los Angeles	2014	Improper sterilization	CRE *K. Pneumoniae*	Duodenoscope	14	2 deaths attributed to CRE infection	(12)
Pittsburgh, PA	2014	Physical defect	MDR- *P. aeruginosa* & Carbapenem-resistant *K. pneumoniae*	Bronchoscope	33	10 deaths	(11)

Among other examples, human error and lack of automatic processes has led to sterilization failure when healthcare workers do not comply with specific guidelines established by hospitals, manufacturers, and regulatory agencies. Outbreaks have been linked to failures of a variety of sterilization methods applied to critical devices to prevent such risks (53, 54). Additionally, medical device design failure, improper storage or maintenance, steam-sterilization malfunction, and other non-human factors may lead to the contamination of medical devices (55).

### Biofilm Formation and Resistance on Medical Devices

A biofilm is a resistant microbial community in combination with a complex matrix of extracellular polymeric substances (EPS), such as proteins and/or polysaccharides, that are needed to attach to the surface (56, 57). The biofilm acts as a protective layer enabling microbes to survive for months under harsh environmental conditions (such as artificial sterilization methods), thus, biofilms may act as microbial reservoirs for recurrent contaminants in both biological and abiotic environments (58, 59). Biofilm formation is initiated through quorum sensing: that is, when a community of bacteria begins to orchestrate chemical communication to interpret changes in their surrounding environment and local cell density. This process is achieved in conjunction with other microbiota within the community to modify gene expression for the production of a variety of adhesion and stress-resistant genes (60). Electron microscopy or other high-powered microscopy techniques are required to view adhesive biofilms within air or water channels of GI endoscopes (61).

Most biofilms in urinary catheters lead to urinary tract infections (UTIs), where multiple bacterial species live in symbiosis. Generally, catheter-associated biofilms can develop both within and outside of urinary catheters (62). These polymicrobial biofilms can promote both resistance and virulence phenotypes through synergistic coexistence and thus increase the threat associated with these microbes (56). Specifically, polymicrobial biofilms such as those found in the disorder of bacterial vaginosis may contribute to the recurrence of catheter-associated UTIs in females. Moreover, the specific composition of catheter-associated polymicrobial biofilms varies greatly. For example, one study identified *Enterococcus faecalis, Escherichia coli*, and *Klebsiella pneumoniae* as the most prevalent species in polymicrobial biofilms, while other studies identified *Enterococcus* spp., *Pseudomonas aeruginosa*, and *Pseudomonas mirabilis* as the most prevalent (63, 64). The association between the compositions of biofilms and various disease states is still unknown, further knowledge can reduce the severity of medical-device-associated infections. Therefore, to mitigate the risk of debilitating MDR biofilm infections from urinary catheters, it is essential for clinicians to employ catheters only when necessary (65).

### Biofilm-Associated Outbreaks

In 1972, it was recognized that an association between medical device infection and biofilms does exist on a wide range of polymeric devices. This has led to the new term “polymer associated infections” (14). The reliable “gold standard” of culturing microbiota that cause recurrent infections may not be possible when communities initiate biofilm formation on medical devices. When a pathogen is exposed to antibiotics or other stressors, it may induce biofilm formation and become viable, but non-culturable, which prevents successful detection of the indicative organism (66). Biofilms not only protect microbes and become a chronic health threat to patients but prompt another significant threat due to their inherent tolerance and resistance to antimicrobials, driving recurrent biofilms and outbreaks associated with medical devices. For example, in a recent study comparing the biofilm formation of a variety of *Acinetobacter* spp. on dry surfaces in hospital environments, such as catheters or glass/plastic, *A. baumannii*-produced biofilms show a greater potential to survive under stressful conditions than non-biofilm-forming species within the same genera (67).

Another emerging MDR pathogen in the hospital setting is *Corynebacterium* spp. which is linked to hospital environments or the normal human skin flora (68). *Corynebacterium striatum specifically* is typically isolated from 50% of immunocompromised patients or individuals who received several sequelae of antibiotics during the course of their treatments (69–71). Additionally, *C. striatum* has been isolated from endotracheal tubes, catheters, and surgical wound wires, illustrating the wide spectrum of medical devices that this organism can thrive upon (72, 73). Generally, the potential for a pathogen to colonize and adhere to both hydrophobic and hydrophilic medical devices, such as catheters, is an important factor in outbreaks associated with medical devices. Generally, biofilm forming microbiota are also able to adhere on various implantable medical devices which employ a wide-spectrum of biomaterials; this highlights the diversity of pathogens thrive in the harshest of human environments (74).

Recently, a study investigated the roles of patient proteins, environmental constraints, and pathogenic factors of *C. striatum* in the onset of biofilms on medical devices (75). The authors found that *C. striatum* was able to persist on polyurethane catheters and develop mature biofilms over time, which was assessed through the application of scanning electron microscopy. The study determined that diverse strains had differing biofilm potential on medical devices. Specifically, the presence of the human protein *Fbg*, which is used in coagulation cascade during inflammation or stress responses, conditioned *C. striatum* and increased the potential for this pathogen to create a biofilm on medical devices (75). *Fbg* uptake has been employed by numerous other hospital-associated pathogens, such as *Corynebacterium diphtheriae, Staphylococcus aureus*, and *Streptococcus suis*, which form cross-bridging with *Fbg* to maintain a biofilm (76–78).

## Risk of Transmission

Microbial contamination of sterile devices is always an inherent risk when using critical medical devices. Specifically, about half of nosocomial infections within a hospital can be associated with microbial transmission or contamination of medical devices (79). To counteract the risk of transmission across patients, most health care facilities maintain high standards of training for medical personnel involved with the disinfection and sterilization process. Therefore, it is essential to ensure adherence to protocols for cleaning, disinfection, and sterilization of medical devices to mitigate infections. For example, *Acinetobacter* spp are ubiquitous microbiota known to cause hospital-acquired infections such as pneumonia, UTIs, bacteraemia, and meningitis in immunocompromised patients (80, 81). The opportunistic nature of *Acinetobacter* spp illustrates the potential of a variety of microbiota to survive and thrive under numerous environmental challenges. Specifically, *A. baumannii* has been identified as a significant threat in the contamination of medical devices (82, 83).

### Risk of Sterilization Failure

Failures in sterilization for the repurposing of critical medical devices may be due to human error, in addition to a variety of misinterpretations surrounding the rationale and importance of high-level sterilization for reprocessing. In September 2015, the CDC announced a health advisory alert for healthcare facilities, to comply with medical device repurposing recommendations to mitigate and control hospital-acquired infections. This specific report called for healthcare facilities to enforce requests for sterilization and to correct workers who were not compliant with critical medical device sterilization processes (84). This is ultimately necessary, as the lack of compliance and standardized recommendations for reprocessing critical medical devices is a significant threat to individual patient safety, in addition to posing a risk of outbreaks that can impact numerous patients directly and indirectly (85). To ensure the safe and effective use of reusable devices while avoiding sterilization failure, the FDA recommends reprocessing instructions in its guidance document (17) for devices falling into any of four categories:

i. *Reusable medical devices initially supplied as sterile to the user and requiring the user to reprocess (i.e., clean and disinfect or sterilize) the device after initial use prior to the subsequent patient use*.
ii. *Reusable medical devices initially supplied as non-sterile to the user and requiring the user to process (i.e., clean, clean and disinfect, or clean and sterilize) the device for initial use, as well as to reprocess the device after each use*.
iii. *Reusable medical devices intended to be reused only by a single patient and intended to be reprocessed between each use*.
iv. *Single-use medical devices initially supplied as non-sterile to the user and requiring the user to process the device prior to its use*.

Regardless of efforts from regulatory agencies, sterilization failures appear to be unavoidable. One of the largest cases of sterilization failure was due to the distribution of inactivated glutaraldehyde disinfectants to 60 hospitals in Belgium. Following this large-scale failure, it was assessed that 34,879 patients were involved in a suspected outbreak, which led to screening of 25,589 patients for both Hepatitis B and C viruses (86). In most cases, inconsistencies in sterilization happen due to human errors during the numerous steps toward proper sterilization and reprocessing, resulting in outbreaks globally (10, 27, 87). Variable factors in the sterilization process include but are not limited to water quality, type of detergent used, amount of time the device interacts with the sterilization product (such as autoclave, in terms of temperature and pressure), drying time, and the sanitary technician's knowledge of sterilization risk management at the time of sterilization (53). Sterilization failure for critical medical devices may leave susceptible patient groups with virulent and potentially recurrent MDR infections. The achievement and maintenance of critical medical device sterilization is ultimately necessary to mitigate incidents and provide a high quality of care to patients. Table 3 describes the global occurrence of endoscope-associated outbreaks due to faulty reprocessing of medical devices and resulting clinical outcomes.

### Environmental Transmission

Control of invasive pathogens that cross-contaminate medical devices from patients or hospital environments has become a major cornerstone in healthcare safety and regulatory risk assessments. The hospital environment plays a significant role in the transmission of contaminants on sterile medical devices if proper precautions are not observed. In the 1970's the CDC suggested that routine culturing of the patient environment was not necessary; at that time the link from air and environmental surfaces to nosocomial disease outcomes was not established (88). Rather, up until 1987 most hospital isolations were used as a precautionary step prior to outbreaks and focused on the diagnosis of infected patients instead of preventing outbreaks or the contamination of medical devices associated with the patient environment (89, 90). There is a lack of regulatory oversight for environmental nosocomial infections and regulations that consider the whole hospital environment in the sterilization of critical medical devices.

Approximately half of healthcare-associated infections of immunocompromised patients are pathogens that are identified either in the environment or through the natural microbial flora in humans. Many of these infections are associated with *Corynebacterium* spp. an emerging MDR pathogen (68, 75). Sterilized medical devices can be contaminated by water for cleaning, health care workers' skin, biofilm formation on surfaces, and other means (41, 42, 67). Half of all hospital-acquired infections of microbial origin can be linked to medical devices (79). Recently, it was demonstrated that an outbreak of the MDR pathogen *Klebsiella oxytoca* was transmitted to newborn children and led to nosocomial infections; the transmission source was a hospital-grade washing machine used to clean clothes in which the pathogen was later isolated from the machine (91). This study indicates a causative, but indirect relationship between hospital patients and the hospital environment.

### MDR Outbreaks Associated With Flexible Critical Medical Devices

In general, MDR pathogens that colonize and persist on critical medical devices are a dynamic problem with the potential to impact a wide variety of medical devices. As with all aspects of medicine, prevention must be a priority to mitigate MDR outbreaks. Outbreaks can be linked to a lack of manual cleaning or brushing, application of contaminated endoscope accessories (such as camera sheaths or elevator shafts), resistance of microbiota to disinfection protocols, and the use of disinfectants that have not been properly processed, thus leading to low efficacy in elimination of microbiota (13, 53, 85, 86, 92).

Several types of medical devices employ flexible endoscopes for a variety of applications such as endoscopic retrograde cholangiopancreatography (ERCP), cystoscopy of the urethral opening, and bronchoscopy of the lungs. The Emergency Care Research Institute has declared that the “inadequate cleaning of flexible endoscopes before disinfection can spread deadly pathogens” as one of the top Health Technology Hazards for 2016 (93). In 2018, the same institute named “failure to consistently and effectively reprocess flexible endoscopes” as the second most important Health Technology Hazard (94).

Since the initial case of a Swedish national returning from India with a urinary tract infection caused by New Delhi metallo-β-lactamase (NBM-1) *K. pneumoniae*, numerous cases of MDR bacteria leading to infections have been identified globally (23). Therefore, there is an urgent need to provide recommendations in established guidelines (17, 95, 96) for the sterilization of flexible endoscopes, in addition to requirements for cleaning, then high-level disinfection.

### Duodenoscope-Mediated Transmission

Duodenoscopes are flexible medical devices typically used in the treatment and diagnosis of several regions within the GI tract, including the small intestines, pancreas, and bile ducts. Because duodenoscopes travel through the intestines of patients and are reused after procedures, there is risk of cross-contamination by the exchange of bacterial load between patients. Due to the complexity in design that enables duodenoscopes to readily move throughout the GI system, these devices are difficult to clean and can be contaminated by MDR bacteria during intended use. MDR outbreaks linked to duodenoscopes are one of the most common types of outbreaks surrounding medical devices. Contamination of this flexible devices typically occurs between the numerous small working parts for fine movements such as joints, elevator shaft, moveable forceps, and camera sheath attachments. The device accessories are difficult to access for cleaning, imposing greater challenges in high-level disinfection procedures (25, 26, 97).

Duodenoscopes are classified as semi-critical medical devices that require a certain standard of cleaning (17); yet, the endoscope can quickly change to critical status depending on the procedure, such as in extraction of biopsy samples (26). In a survey of 116 hospitals in the US, a 6% rate of infection was reported due to endoscopy (98). Specifically, carbapenem-resistant outbreaks were noted to have rates of 23–38% infection or colonization on endoscopes following ERCPs (32, 99).

It has been reported that inadequate sterilization of endoscopes used for ERCPs may create reservoirs for MDR bacteria such as *K. pneumoniae*, which can also persist and chronically contaminate duodenoscopes regardless of repeated disinfections (27, 100). These factors may lead to a variety of reported and unreported outbreaks of MDR pathogens. Many outbreaks associated with flexible endoscopes are caused by carbapenem-resistant *Enterobacteriaceae* (CRE) which are resistant to a last-resort class of antibiotics and pose a major threat to patients as an invasive pathogen residing on or within endoscopes. Further CRE outbreaks are still reported even though sterilization facilities report that no mistakes in the reprocessing procedures for reusable devices can be identified (32, 33). In the first major report of CRE associated with GI endoscopes in the US, 38 patients contracted CRE following an ERCP at a hospital (32). Of these 38 patients, only 10 had true clinical infections, while the remaining 28 were found to be colonized by these bacteria without symptoms during a routine surveillance culture. Interestingly enough, a carbapenem-resistant *K. pneumoniae* was also isolated from the terminal end of the ERCP endoscope and was associated with the CRE in the same infection; *K. pneumoniae* was not shown to cause an infection in this case.

### Urogenital Transmission

While it has been long believed that human urine and the urogenital tract are sterile, recent evidence in microbiome studies has suggested that a functional microbial community is present there in both health and disease (101, 102). Endoscopy of the urogenital tract has a high possibility to expose patients to pathogens associated with urinary tract infections (UTIs), such as *Escherichia coli* or *Klebsiella pneumoniae*. In an urogenital endoscopy outbreak, the application of camera sheaths and cleaning methods were inconsistent (13). Interestingly enough, the infection control standards classify video camera heads used for endoscopy as a non-critical device (103), due to the fact that the camera does not typically interact with the patient tissue. There is a lack of standardized guidelines for the control of infections associated with video camera heads, as applied for urology in the UK (13, 103).

Due to misinterpretation of medical device classifications in patient care, there is a lack of standardization in sterilization practices. For example, failed sterilization of a urogenital scope led to an outbreak with the novel strain of MDR NDM-1 *Klebsiella* that impacted 12 patients in July 2010 and led to urosepsis for three patients (13). There is an urgent need to provide recommendations for the standardization of application of interchangeable camera head sheaths for infection control; specifically, the application of single-use sterile sheaths and cleaning with disinfectants that will not damage the camera lens are recommended to prevent cross-contamination across patients (13).

### Bronchoscope-Mediated Transmission

Bronchoscopes are used to investigate and retrieve specimens from the lungs via a suction channel. There are two types of bronchoscopes: rigid and flexible. The latter typically requires use of a video camera eyepiece that is prone to contamination between uses. There were 48 outbreaks recorded between the years 1970 and 2012 related to cross-contaminations and bronchoscopy (5, 97). In recent years, numerous outbreaks related to the cross-contamination of both carbapenem-resistant *K. pneumoniae* and MDR *P. aeruginosa* have been linked to bronchoscopes (3, 104, 105).

Generally, outbreaks associated with MDR bacteria on bronchoscopes are under-reported (5). Due to the extensive outbreaks associated with improper cleaning and contamination of reusable bronchoscopes, the FDA published a communication to highlight the risks of such contamination in September 2016 (34). For example, when a bronchoscope marketed as “single-use” was misinterpreted as “single-patient,” it initiated an outbreak of opportunistic *P. aeruginosa* that led to nosocomial pneumonia and both bloodstream and respiratory infections. After 24 h, both *S. aureus* and *K. pneumonia* were isolated from the bronchoscopes; both may prove fatal to immunosuppressed or critically ill patients (8). In this case study, the “single-patient” bronchoscopes were repurposed; after over 48 h, seven (35%) of the bronchoscopes had pathogens considered to be of high risk to critically ill patients.

In another outbreak, failure to effectively disinfect the bronchoscope and remove microbial biofilms from the device was reported (10). The sterilization protocol prior to the outbreak requires either cleaning the bronchoscope surfaces using 70% ethanol or a diluted detergent that contained enzymes, followed by immersion in ortho-phthalaldehyde solution (106, 107). Due to delicate parts in the bronchoscope, the initial disinfection protocol alone is unable to remove the biofilms (108). This case study highlights that some forms of high-level disinfection are not sufficient to prevent outbreaks. The outbreaks subsided only when the protocol added a pre-cleaning step to reduce any organic material as well as both hypochlorous acid solution and ETO weekly sterilization (10, 109).

## Regulatory Recommendation of Sterilization and Reprocessing

Due to the emergence of MDR bacteria and biofilms on different classes of medical devices, we recommend that requirements for terminal sterilization and high level-disinfection of reprocessable medical devices should be unified globally, devices should be critically evaluated before clearance into the market for patient use. It is important to redefine Spaulding's initial classification for semi-critical and critical medical device categories for the 21st century. This would require the reclassification and reassessment of numerous medical devices, in addition to accessories or attachments that are readily contaminated between uses.

Depending on the materials used in medical devices, there are numerous ways to sterilize them, though these technologies also have drawbacks depending on the function and structure of the device to be sterilized. These drawbacks can jeopardize the safety and efficacy of the medical devices (85, 110); for example, EtO residues can be toxic to individuals (111).

Among the most common ways to sterilize medical devices is to use a dry or wet autoclave, which employs both heat and pressure to eliminate microbiota. Typically, the steam autoclave is non-toxic to users and rapidly eliminates microorganisms and their spores. However, some medical devices are heat sensitive, and/or residual water from the steam may damage them due to rusting. Additionally, moisture from steam may generate an environment for microbiota to grow if not properly dried (112).

Sterilization with hydrogen peroxide (H_2_O_2_) gas could be a method compatible with most medical devices. Vaporized H_2_O_2_ or H_2_O_2_ with ozone can also be employed; both take considerably longer than hydrogen peroxide alone (28 vs. 55 min), and both lack evidence related to clinical usage (such as compatibility with medical device materials or resistance on materials). Further, there is a lack of data describing the hypothesized microbicidal effects.

To date, 100% EtO is the gold-standard method for high-level sterilization of medical devices. EtO has been employed to control outbreaks by sterilizing contaminated medical devices, though it is not common in most hospitals and is a long-term intervention that is time-intensive (10, 85, 109). The EtO method has other drawbacks: it typically cannot be employed in a clinical setting for numerous reasons, which include long processing time and toxic or carcinogenic residues (113). To mitigate exposure to EtO residues on medical devices, it is necessary to allow EtO-treated medical devices to aerate for an additional period of time (~12–15 h) (85).

While patients are notified of infections resulting from human error during reprocessing of a device, there are no criteria for reporting to regulatory bodies, which is essential for establishing regulatory standards. Any outbreak due to contaminated medical devices should be unacceptable. The scientific threshold should be established by specific benchmarks, scientific readings, etc. While risks are inherent during surgical procedures, they are modulated by a risk-benefit ratio which requires the mitigation of risk and benefits for both the patient and hospital efficacy (53, 114).

Overall, health care policies are needed to identify infection risks based on use of semi-critical and critical medical devices. Further, the possibility of infection from non-critical medical devices cannot be denied, users must be equipped with properly reviewed instructions for use from regulatory agencies (115). In addition, it is essential to establish control methods to mitigate processing errors to prevent patient exposure to contaminated medical devices. Before semi-critical and critical reusable devices are used on patients, there is a great need to establish appropriate reprocessing procedures with trained technical staff and device-specific requirements for a high margin of safety and efficacy.

## Conclusion

The potential for medical devices to be colonized by MDR pathogens and their biofilms is a serious challenge for patient safety and public health. More so, several outbreaks associated with different types of medical devices have been linked to improper sterilization and reprocessing and could have been prevented if regulations were followed. This article echoes the FDA's established guidance for sterilization and reprocessing of medical devices, along with recommendations to control the numerous routes of transmission and enforce appropriate sterilization protocols for differing medical devices. There is a need to revisit not just the sterilization strategy for classified medical devices, but also the circumstances in which the devices are used, so that risks of contamination can be mitigated based on appropriate user conditions.

## Author Contributions

JJ-S wrote the manuscript. OS planned, reviewed, and wrote the manuscript. All authors contributed to the article and approved the submitted version.

## Conflict of Interest

OS was employed by the company TSG Consulting and The Johns Hopkins University at the time manuscript was prepared. The remaining author declares that the research was conducted in the absence of any commercial or financial relationships that could be construed as a potential conflict of interest.
